# Prognostic Significance of p53 Immunohistochemical Expression and Molecular Characterization of TP53 Variants in Triple-Negative Breast Cancer After Neoadjuvant Chemotherapy

**DOI:** 10.3390/diagnostics16193229

**Published:** 2026-10-06

**Authors:** Juan Ramón Berenguer-Marí, Luis Álvarez, Julia Sierra-Roca, Raquel Bosch-Romeu, Irene Tadeo-Cervera, Juan Pardo, Antonella Locascio, Juan Bautista Laforga, Antonio Falcó, Juan Manuel Gasent-Blesa, Joan Climent

**Affiliations:** 1 Department of Medical Oncology, Hospital de Denia, 03700 Alicante, Spain; berenguer_juamar1@gva.es; 2 PASAPTA Department, Veterinary School, Universidad Cardenal Herrera-CEU, CEU Universities, Alfara del Patriarca, 46115 Valencia, Spain; luis.alvarezfernandez@uchceu.es (L.Á.); irene.tadeo@uchceu.es (I.T.-C.); gasent_joa@gva.es (J.M.G.-B.); 3 Biomedical Research Institute, Universidad Cardenal Herrera-CEU, CEU Universities, Alfara del Patriarca, 46115 Valencia, Spain; 4 Department of Obstetrics and Gynecology, La Ribera Salud, 46015 Sueca, Spain; m.j.sierraroca@hotmail.com; 5 Department of Mathematics, Physics and Technological Sciences, Universidad Cardenal Herrera-CEU, CEU Universities, 46115 Valencia, Spain; raquel.boschromeu@uchceu.es (R.B.-R.); juaparal@uchceu.es (J.P.); afalco@uchceu.es (A.F.); 6 ESI International Chair@CEU-UCH, Universidad Cardenal Herrera-CEU, CEU Universities, Alfara del Patriarca, 46115 Valencia, Spain; 7 School of Health Sciences, Universidad Cardenal Herrera-CEU, CEU Universities, Alfara del Patriarca, 45115 Valencia, Spain; antonella.locascio@uchceu.es; 8 Department of Pathology, Hospital de Denia, 03700 Alicante, Spain; jblaforga@gmail.com; 9 ECMOR Chair@CEU-UCH, Universidad Cardenal Herrera-CEU, CEU Universities, Alfara del Patriarca, 46115 Valencia, Spain

**Keywords:** triple-negative breast cancer, TP53, p53, immunohistochemistry, neoadjuvant chemotherapy, pathological complete response, prognosis, longitudinal TP53 changes

## Abstract

**Background:** The prognostic significance of p53 immunohistochemical expression in triple-negative breast cancer (TNBC) remains controversial, particularly following neoadjuvant chemotherapy (NAC). The relationship between p53 immunohistochemical expression, TP53 sequence variants, and clinical outcome following neoadjuvant chemotherapy remains incompletely understood. This study evaluated the relationship between p53 expression, TP53 variant profiles, longitudinal changes in TP53 variant detection and allele frequency, and clinical outcome in patients with TNBC treated with NAC. **Methods:** Forty-three patients with TNBC treated with NAC were retrospectively analyzed. p53 immunohistochemistry performed on tumor samples collected before NAC was evaluated using binary (<10% vs. ≥10%) and three-tier (<10%, 10–50%, >50%) classifications. We performed targeted sequencing on tumors before treatment and available matched post-treatment samples to characterize TP53 variants and longitudinal changes in variant detection and variant allele frequency (VAF). We assessed associations with pathological complete response (pCR), recurrence, disease-free survival (DFS), and overall survival (OS). **Results:** High p53 expression assessed before NAC (≥10%) was significantly associated with improved DFS and OS among patients achieving pCR, whereas no significant prognostic association was observed in patients with residual disease. This differential association according to pathological response was supported by formal interaction analyses. TP53 variant status and baseline variant burden were not significantly associated with clinical outcome, and patient-level TP53 VAF did not significantly differ among prognostic groups. Longitudinal TP53 sequencing identified shared and timepoint-specific variants and changes in VAF between baseline and post-treatment samples. These molecular findings were considered exploratory and descriptive. **Conclusions:** p53 immunohistochemical expression assessed before NAC showed prognostic associations that differed according to pathological response, whereas TP53 sequencing provided descriptive molecular characterization and did not demonstrate independent prognostic stratification. These findings support further investigation of p53 IHC as a potential response-contextual prognostic marker and provide exploratory insight into longitudinal changes in TP53 variant detection and VAF following NAC.

## 1. Introduction

Triple-negative breast cancer (TNBC) remains one of the most clinically challenging subtypes of breast cancer, accounting for approximately 15–20% of all newly diagnosed cases [1]. Defined by the absence of estrogen receptor (ER), progesterone receptor (PR), and human epidermal growth factor receptor 2 (HER2) expression, TNBC is characterized by marked biological heterogeneity, aggressive clinical behavior, and limited therapeutic options [1]. Unlike other breast cancer subtypes, targeted therapies are available for only a subset of patients, making systemic chemotherapy the cornerstone of treatment for localized disease [1]. In this setting, neoadjuvant chemotherapy (NAC) has become the standard of care, providing not only tumor downstaging but also an in vivo assessment of chemosensitivity [2]. Although patients achieving a pathological complete response (pCR) generally experience excellent long-term outcomes, a substantial proportion either fail to achieve pCR or subsequently relapse despite an apparently favorable pathological response [2,3]. This marked variability in clinical outcome highlights the need for robust biomarkers capable of improving prognostic stratification and identifying patients at risk of recurrence beyond conventional clinicopathological variables.

Among the genomic alterations characterizing TNBC, TP53 is by far the most frequently mutated gene, being altered in approximately 70–80% of tumors [4,5]. As the central regulator of genomic integrity, p53 orchestrates multiple cellular processes, including DNA damage sensing, cell-cycle arrest, apoptosis, senescence, DNA repair, and metabolic adaptation to cellular stress [6,7]. Consequently, disruption of the TP53 pathway represents one of the defining molecular events in TNBC biology and has been implicated in tumor initiation, genomic instability, metastatic progression, and response to cytotoxic therapy [4,5,6,7]. However, despite its unquestionable biological importance, the clinical significance of TP53 alterations remains incompletely understood. Numerous studies have attempted to correlate TP53 mutations or p53 protein expression with patient outcome, yet the results have often been inconsistent, limiting their incorporation into routine clinical decision-making [8,9,10,11,12].

In parallel, increasing evidence suggests that therapeutic response in TNBC cannot be fully characterized through analysis of tumors collected before NAC alone. Paired molecular studies have demonstrated differences in variant detection and molecular profiles between baseline tumors and residual disease after treatment [12,13,14,15]. Although several genomic studies have characterized residual TNBC following NAC [13,14,15], relatively few have examined longitudinal TP53 variant changes alongside p53 immunohistochemical expression and clinical outcome.

In the present study, we evaluated p53 immunohistochemical expression and TP53 sequence variants in a retrospective cohort of patients with TNBC treated with neoadjuvant chemotherapy. We investigated the prognostic significance of p53 expression according to pathological response and characterized TP53 variants at baseline and longitudinally in available paired pre- and post-treatment tumor samples.

## 2. Materials and Methods

We conducted a retrospective analysis of 43 patients diagnosed with triple-negative breast cancer (TNBC) treated with neoadjuvant chemotherapy. Patient and sample availability for p53 IHC, baseline and post-treatment TP53 sequencing, paired longitudinal analysis, and p53-stratified survival analysis are summarized in Supplementary Figure S1. Pathological complete response (pCR) was defined as the absence of residual invasive carcinoma in the breast and axillary lymph nodes after NAC. Pathological response percentage represented the estimated percentage of tumor response after NAC based on pathological assessment, with 100% indicating complete pathological response and lower values indicating residual tumor. Recurrence was defined as local or distant disease recurrence after NAC. DFS was calculated from the date of diagnosis to the first documented recurrence or, for patients without recurrence, to the date of last follow-up. OS was calculated from the date of diagnosis to death or, for surviving patients, to the date of last follow-up. Patients were classified into four groups according to pathological response and recurrence status: G0R0, G0R1, G1R0 and G1R1, where G0 indicated non-complete pathological response, G1 complete pathological response, R0 absence of recurrence and R1 recurrence. Clinical, pathological, immunohistochemical and molecular data were included for comparative analyses across these groups. This retrospective single-center study included patients with triple-negative breast cancer (TNBC) treated with taxane-based neoadjuvant chemotherapy, with or without anthracyclines, at Hospital de Dénia (Spain) between 2009 and 2019. The study cohort, inclusion criteria, clinicopathological characteristics, treatment protocol, and biospecimen collection have been described previously [16]. The cohort predominantly comprised patients with localized or locally advanced TNBC. Two patients had oligometastatic stage IV disease at diagnosis due to biopsy-confirmed liver metastases and were included because they underwent multimodal treatment with radical intent, including systemic therapy and breast surgery. Distant metastatic disease diagnosed during follow-up was recorded separately from the post-NAC pathological assessment; metastatic disease was considered pathologically confirmed when supported by biopsy of the distant lesion. Only patients with available formalin-fixed paraffin-embedded (FFPE) tumor tissue suitable for molecular and immunohistochemical analyses were included. Specimens selected for molecular analysis were required to contain ≥70% tumor cells, as determined by histopathological assessment. Clinicopathological and follow-up information was obtained from the institutional electronic medical records, and tumor staging was performed according to the AJCC TNM classification. The study was approved by the Research Committee of the Dénia Health Department (2 June 2018. Reference PI00018) and conducted in accordance with the Declaration of Helsinki. Written informed consent was obtained from all living participants, and all data were anonymized before analysis.

### 2.1. p53 Immunohistochemistry and Survival Analysis

p53 immunohistochemistry was performed on FFPE tissue sections using a ready-to-use monoclonal mouse anti-human p53 antibody (clone DO-7, FLEX RTU; Dako/Agilent Technologies, Santa Clara, CA, USA). Following deparaffinization and rehydration, heat-induced antigen retrieval was performed in citrate buffer (pH 6.0). Immunoreactivity was visualized using a peroxidase-based detection system with diaminobenzidine (DAB) as the chromogen.

p53 expression was quantified as the percentage of tumor cells showing nuclear staining. Two complementary classification schemes were applied. For the binary analysis, tumors were classified as having low p53 expression (<10% positive tumor cells) or p53 positivity (≥10%), using 10% as the predefined cutoff. To further explore potential heterogeneity within p53-positive tumors, expression was additionally categorized into three groups according to the percentage of positively stained tumor cells: <10% (low), 10–50% (intermediate), and >50% (high expression). The 10% and 50% thresholds were selected because they are among the most frequently used cutoffs in previous breast cancer studies, although no universally validated cutoff for p53 IHC has been established [11].

Disease-free survival (DFS) and overall survival (OS) were estimated using the Kaplan–Meier method and compared with the log-rank test. Survival analyses were performed both in the overall cohort and after stratification by pathological response group (G0 vs. G1), using both the binary and three-tier p53 IHC classifications. To formally assess whether the association between p53 expression assessed before NAC and survival differed according to pathological response, regression models including p53 expression (<10% vs. ≥10%), pathological response (pCR vs. residual disease), and their interaction term were fitted. For DFS, Cox proportional hazards regression was used, and the significance of the interaction was assessed using a likelihood-ratio test comparing models with and without the interaction term. Hazard ratios (HRs) and 95% confidence intervals (CIs) for p53 expression were additionally estimated within each pathological response subgroup. For OS, Firth penalized Cox regression was used because no deaths occurred among patients with pCR and p53 expression ≥10%, resulting in complete separation in conventional Cox regression. The p53-by-pathological-response interaction and subgroup-specific HRs and 95% CIs for OS were therefore estimated using Firth penalized Cox regression.

### 2.2. TP53 Variant Analysis and Bioinformatic Pipeline

Genomic DNA was extracted from FFPE tumor tissue using the GeneRead DNA FFPE Kit (QIAGEN, Hilden, Germany) and quantified using a Qubit 3.0 fluorometer with the dsDNA High-Sensitivity Assay. Targeted sequencing was performed using the AmpliSeq Cancer HotSpot Panel v2 (Illumina), which interrogates approximately 2800 hotspot regions across 50 cancer-related genes. Libraries were sequenced on an Illumina MiSeq platform using MiSeq Reagent Kit v2 (Illumina, San Diego, CA, USA). FASTQ files were generated using MiSeq Reporter. Sequence alignment and secondary analysis were performed using the DNA Amplicon Analysis workflow in Local Run Manager v3. Reads were aligned to the UCSC hg19/GRCh37 human reference genome, and the resulting BAM files were used for variant calling with Pisces variant caller v5.2.9.23 (Illumina) in somatic calling mode. Variant calls were exported as genome.vcf.gz files and subsequently imported into BaseSpace Variant Interpreter for filtering, annotation, and interpretation. Single-nucleotide variants, multinucleotide variants, and short insertions/deletions were considered. Variants retained for the revised molecular analyses were required to have a VAF ≥ 2.5%.

Variant coordinates originally based on the hg19/GRCh37 human genome assembly were converted to GRCh38 prior to the updated variant annotation. TP53 variants detected in baseline and post-treatment samples were subsequently annotated using Ensembl Variation BioMart and Ensembl Variant Effect Predictor (VEP), including variant consequence, variant class, existing variant identifiers, population allele frequencies, ClinVar clinical significance, and germline and somatic database information. Variant consequences were classified as missense, truncating, splice, in-frame, synonymous, or other, with missense variants further characterized using UMD TP53 functional annotation [17]. For population classification, European allele frequency (EUR_AF) was prioritized when available; otherwise, the maximum reported population allele frequency (MAX_AF) was used. Variants with an allele frequency ≥5% were classified as population polymorphisms, those with an available frequency <5% as non-polymorphic variants, and variants lacking population frequency information were retained as a separate category.

The association between TP53 variant status and p53 immunohistochemical expression in tumors obtained before NAC was evaluated after excluding variants classified as population polymorphisms. Patients were classified according to the presence or absence of additional TP53 variants other than population polymorphisms. Associations with the binary (<10% vs. ≥10%) and three-tier (<10%, 10–50%, >50%) p53 IHC classifications were assessed using Fisher’s exact test.

For each patient, TP53 variant burden was calculated by counting the number of distinct TP53 variants detected at baseline and post-treatment. VAF was analysed at the variant level and summarized per patient using maximum, mean, and median VAF values. Comparisons of variant burden and VAF across clinical groups were performed using non-parametric tests, including Kruskal–Wallis and Wilcoxon rank-sum tests, as appropriate.

A complete list of TP53 variants, including genomic coordinates, VAF, functional classification, database annotation, and UMD-derived functional information, is provided in Supplementary Table S1. Statistical analyses and downstream processing of the annotated variant data were performed in R (RStudio version ‘2024.12.0.467’) [18].

### 2.3. Paired Longitudinal TP53 Variant Analysis

Paired baseline and post-treatment samples were used to evaluate longitudinal changes in TP53 variant detection and VAF. Variants were classified as shared when detected at both timepoints, baseline-only when detected only before NAC, and post-treatment-only when detected only after NAC. For shared variants, the direction and magnitude of the change in VAF between baseline and post-treatment samples were calculated and evaluated descriptively. Because no prespecified technical or biological threshold for a meaningful longitudinal VAF change was available, variants were not formally classified as increased, decreased, or stable.

Longitudinal VAF changes were visualized using paired VAF plots and spaghetti plots. Variant overlap analyses were performed to compare TP53 alterations across prognostic groups, including the overlap between variants present in responders at baseline and baseline-only variants or shared variants showing numerically lower post-treatment VAF in non-responders without recurrence. VAF values were not adjusted for the exact tumor-cell fraction of individual specimens and were therefore interpreted as relative sequencing measurements rather than estimates of cancer-cell fraction. Functional composition of baseline-only, shared, and post-treatment-only variants was summarized graphically using stacked bar plots and Venn diagrams.

## 3. Results

### 3.1. Clinical and Pathological Characteristics

The study included 43 patients with TNBC treated with neoadjuvant chemotherapy between 2009 and 2019, including two patients with oligometastatic stage IV disease at initial diagnosis. Pathological complete response (pCR) was achieved in 18 patients (41.9%), whereas 25 patients (58.1%) had residual disease following treatment. Baseline p53 IHC was evaluable in 42 patients, including 17 of the 18 patients who achieved pCR. Baseline TP53 sequencing was available for 40 patients, of whom 39 also had evaluable p53 IHC. Post-treatment sequencing was available for 21 patients with residual disease, and 18 had matched baseline/post-treatment sequencing data for longitudinal analysis. Patient and sample availability for each analysis is summarized in Supplementary Figure S1. A comprehensive description of the demographic, clinicopathological, treatment-related, and molecular characteristics of the cohort is provided in our previous report [16] and is summarized in Supplementary Table S2.

### 3.2. Clinicopathological and Molecular Variables Associated with Survival Prognosis

We next evaluated the clinicopathological and molecular variables significantly associated with survival outcomes, with a focus on recurrence and overall survival.

### 3.3. Recurrence and Disease-Free Survival

As expected, the analysis of post-treatment variables revealed that pathological response percentage was significantly lower in patients who relapsed compared with those who remained disease-free (median 50% (10–100) vs. 100% (88–100); *p* = 0.017). Indicators of residual tumor burden also distinguished the two groups: patients with recurrence had a higher number of affected lymph nodes post-treatment (median 0 (0–1.8, mean 1.5 ± 3.0) compared with those without recurrence (median 0 [0–0], mean 0.2 ± 0.7; *p* = 0.034).

### 3.4. Overall Survival and Death

When comparing patients who died with survivors, a complementary set of prognostic variables emerged. At the pre-treatment level, p53 expression was the only factor associated with death, with low p53 expression (<10%) more frequent among deceased patients (58.8% vs. 28.0% in survivors; p = 0.046).

Most variables significantly associated with death were post-treatment pathological variables. The percentage of pathological response was markedly reduced in deceased patients (median 49% [0–58]) compared with survivors (median 100% [90–100]; *p* = 0.0005). Consistently, response status reflected this difference, with non-responders predominating among deceased patients (76.5% vs. 46.2% in survivors; *p* = 0.049). Markers of residual disease burden were also strongly associated with mortality. Deceased patients had a higher number of affected lymph nodes post-treatment (median 0 [0–3], mean 1.9 ± 3.3) than survivors (median 0 [0–0], mean 0.2 ± 0.6; *p* = 0.0027). Similarly, higher T stage post-treatment (41.2% vs. 7.7%; *p* = 0.037), and N-stage positivity post-treatment (58.8% vs. 11.5%; *p* = 0.0036) were all significantly associated with death. Lymph-node positivity was also significantly more frequent among deceased patients (47.1% vs. 7.7%; *p* = 0.0072). Taken together, these findings indicate that poorer pathological response and greater residual disease burden after NAC were associated with recurrence and mortality. Low baseline p53 expression was additionally more frequent among patients who died, supporting further evaluation of its potential prognostic relevance.

### 3.5. Clinicopathological and Molecular Variables Associated with Pathological Complete Response

To better understand the clinicopathological and molecular determinants of therapeutic efficacy, patients were stratified according to their pathological response to neoadjuvant chemotherapy and disease recurrence during follow-up. This combined classification generated four distinct subgroups: G0R0 (non-responders without recurrence), G0R1 (non-responders with recurrence), G1R0 (responders without recurrence), and G1R1 (responders with recurrence). This integrative approach allowed the joint evaluation of treatment sensitivity and long-term outcome, identifying patients who achieved durable remission after pCR (G1R0) and those with early relapse despite response (G1R1), as well as resistant tumors with (G0R1) or without recurrence (G0R0). Comparative analyses across these four subgroups were then performed to assess differences in clinicopathological variables and TP53 gene-specific variants, providing a comprehensive overview of the biological and clinical features associated with pathological complete response and recurrence risk in TNBC.

### 3.6. Prognostic Impact of p53 Expression Within Response Groups

**Figure 1:**
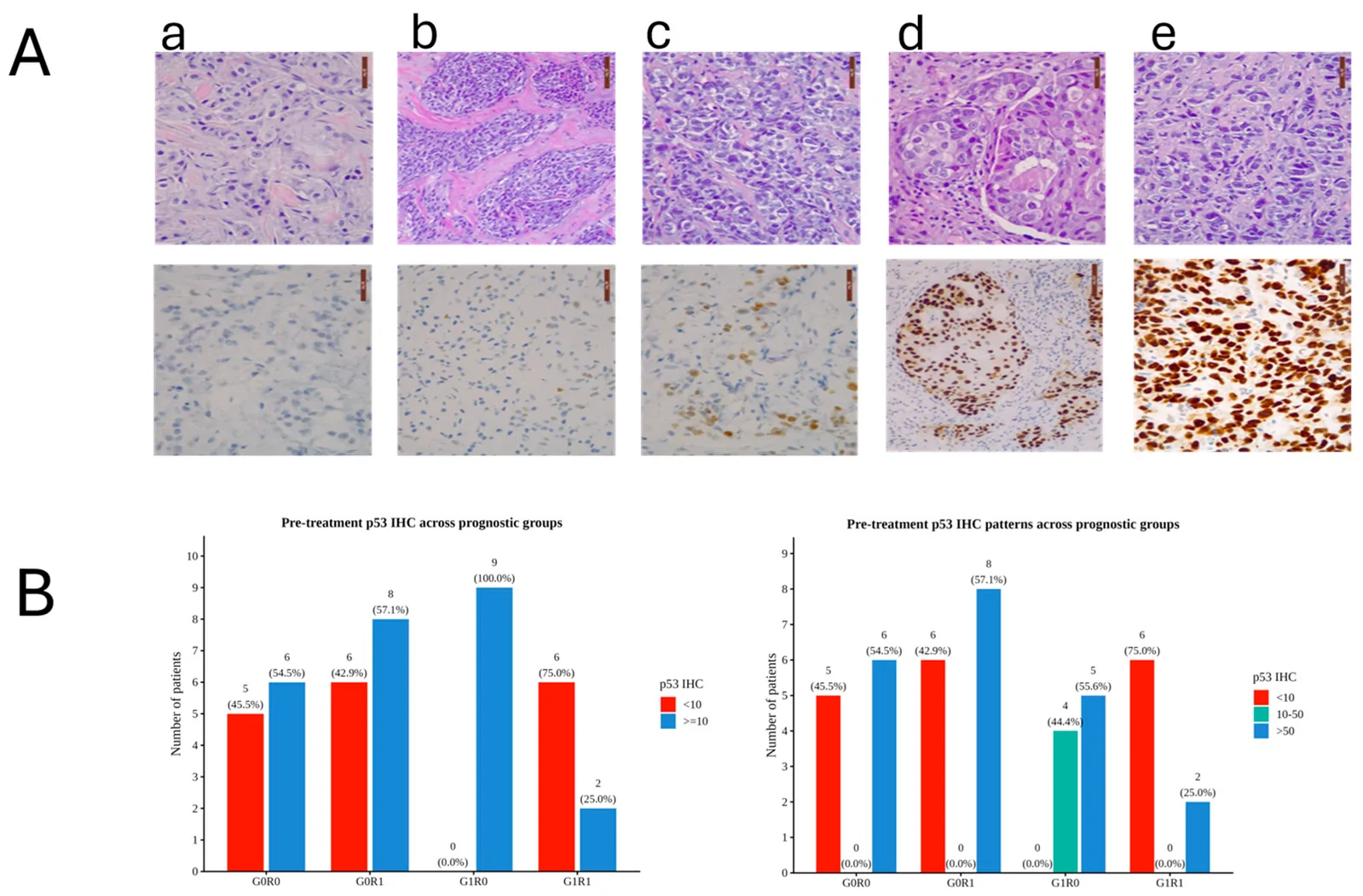
(**A**) Representative histological and p53 immunohistochemical findings in TNBC tumors showing different levels of p53 expression. (**Upper row**): hematoxylin and eosin (H&E)-stained sections; (**Lower row**): corresponding p53 immunohistochemical (IHC) staining. Representative paired images illustrate complete absence of p53 staining (**a**,**b**), low/intermediate expression (~20%) (**c**), high expression (~80%) (**d**), and diffuse expression approaching 100% of tumor cells (**e**). (**B**) Distribution of p53 expression across prognostic subgroups defined by treatment response (G0 = non-response, G1 = response) and recurrence status (R0 = no recurrence, R1 = recurrence). (**Left panel**): binary classification (<10% vs. ≥10%). (**Right panel**): three-group classification (<10%, 10–50%, >50%). Non-responder groups (G0R0 and G0R1) showed mixed distributions dominated by either low or strongly positive tumors, whereas the responder/non-recurrence group (G1R0) lacked tumors with <10% expression and showed enrichment of intermediate-to-high expression patterns. In contrast, the responder/recurrence group (G1R1) was predominantly composed of tumors with low p53 expression (<10%).

Representative histological and immunohistochemical images illustrating the spectrum of p53 expression observed in the tumors are shown in Figure 1A, ranging from complete absence of nuclear staining to intermediate and diffuse strong p53 expression. We next analyzed p53 immunohistochemical expression across the four prognostic subgroups defined by pathological response and recurrence status (G0R0, G0R1, G1R0, and G1R1). Distinct p53 expression patterns emerged among groups (Figure 1B).

Using the binary classification (<10% vs. ≥10%), non-responders without recurrence (G0R0) showed a relatively balanced distribution, with 5 of 11 patients (45.5%) exhibiting negative p53 expression (<10%) and 6 (54.5%) showing positive p53 expression (≥10%). A similar distribution was observed in non-responders with recurrence (G0R1), where 6 of 14 patients (42.9%) displayed negative expression and 8 (57.1%) positive expression.

In contrast, all patients achieving pathological response without recurrence (G1R0, 9/9) exhibited positive p53 expression (≥10%). Conversely, the responder group that subsequently relapsed (G1R1) showed the opposite pattern, with negative p53 expression predominating in 75.0% of cases (6/8), whereas only 25.0% (2/8) retained positive expression. These differences were statistically significant (*p* = 0.0117, Fisher’s exact test), indicating that p53 expression patterns may distinguish patients achieving durable remission from those relapsing despite treatment response.

To further refine these patterns, p53 expression was additionally stratified into three categories: negative (<10%), intermediate (10–50%), and strong overexpression (>50%) (Figure 1B). This analysis revealed that the favorable responder/non-recurrence group (G1R0) was characterized by the absence of completely negative tumors and by enrichment of intermediate-to-strong p53 expression patterns. Specifically, 44.4% of G1R0 tumors showed intermediate expression (10–50%), while 55.6% exhibited strong overexpression (>50%). By contrast, the responder/recurrence group (G1R1) was dominated by p53-negative tumors (<10%, 75.0%), with no tumors displaying intermediate expression.

Interestingly, intermediate p53 expression (10–50%) was absent in both non-responder groups (G0R0 and G0R1), where tumors segregated almost exclusively into either completely negative (<10%) or strongly positive (>50%) patterns. These findings suggest that intermediate p53 expression may be associated with a more favorable biological phenotype and durable disease control after pathological response.

**Figure 2:**
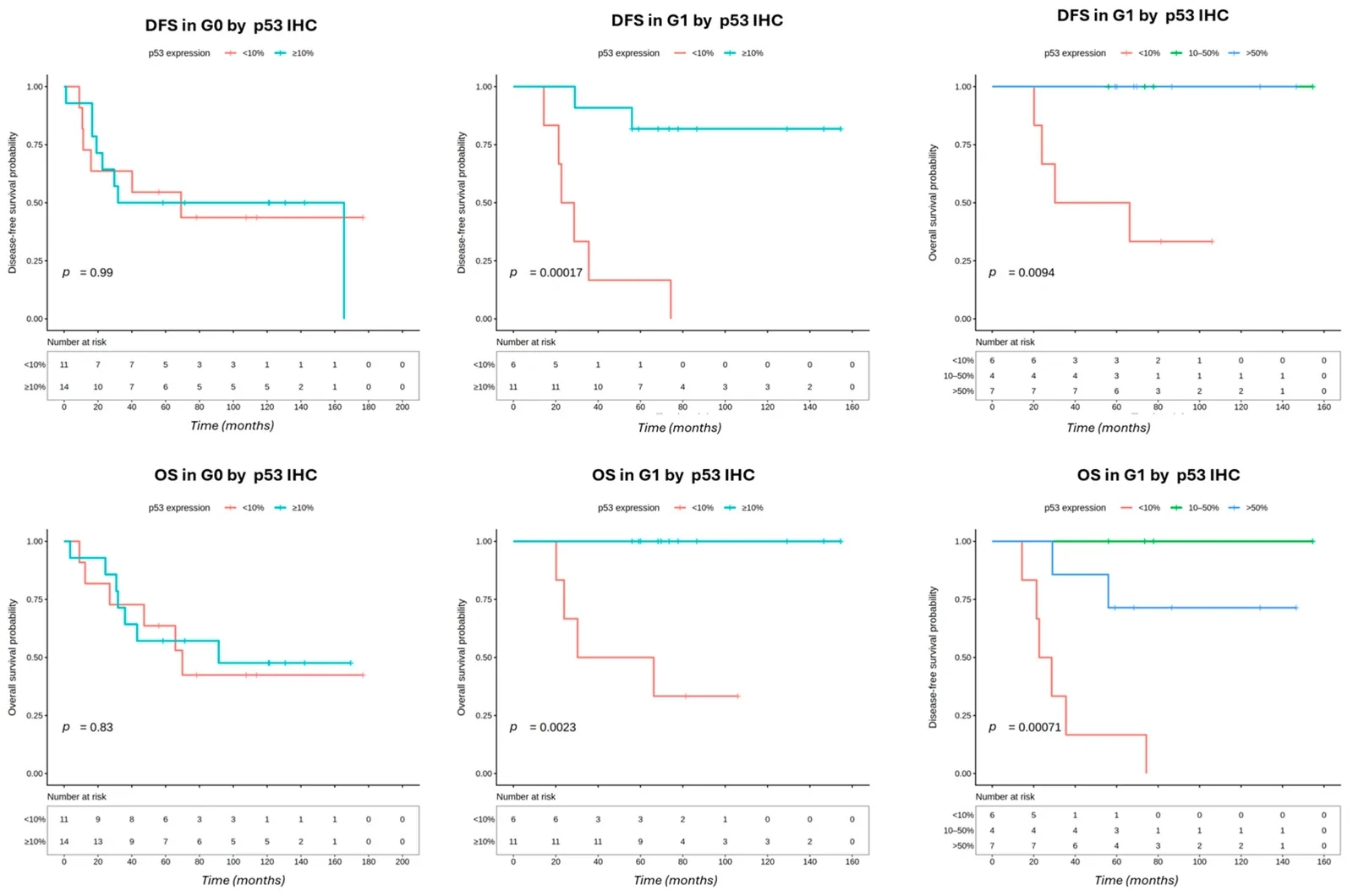
Kaplan–Meier survival analyses according to p53 immunohistochemical expression and pathological response. Disease-free survival (DFS; **upper row**) and overall survival (OS; **lower row**) according to p53 expression assessed before NAC. (**Left panels**) show patients with residual disease (G0) stratified using the binary p53 classification (<10% vs. ≥10%). (**Middle panels**) show patients achieving pathological complete response (G1) using the same binary classification. (**Right panels**) show patients achieving pCR (G1) stratified using the three-tier p53 classification (<10%, 10–50%, and >50%). Log-rank *p*-values are shown in each panel. Numbers at risk are provided below each Kaplan–Meier curve.

We next assessed the prognostic significance of p53 expression within chemotherapy response-defined subgroups (G0 = non-responders, G1 = responders) using Kaplan–Meier analysis for disease-free survival (DFS) and overall survival (OS) (Figure 2).

In the G0 subgroup (non-responders), neither DFS nor OS differed according to binary p53 status (<10% vs. ≥10%). Kaplan–Meier curves for patients with negative and positive p53 expression were nearly superimposable, and log-rank tests confirmed the absence of significant differences (DFS: χ^2^ = 0.00009, *p* = 0.992; OS: χ^2^ = 0.046, *p* = 0.83). Because the intermediate-expression category (10–50%) was absent in non-responder tumors, three-group stratification did not provide additional information in this subgroup.

In contrast, among responders (G1 subgroup), p53 expression strongly stratified survival outcomes. Using the binary classification, patients with positive p53 expression (≥10%, n = 11) showed prolonged disease-free and overall survival compared with patients with negative expression (<10%, n = 6). Kaplan–Meier curves showed clear separation between groups, with highly significant differences by log-rank test (DFS: χ^2^ = 14.09, *p* = 0.00017; OS: χ^2^ = 9.33, *p* = 0.0023).

Importantly, three-group stratification further refined prognostic discrimination within responders. Patients with intermediate p53 expression (10–50%) showed the most favorable clinical course, maintaining prolonged DFS and OS throughout follow-up. Tumors with strong overexpression (>50%) also showed favorable outcomes, although slightly less pronounced than the intermediate-expression subgroup, whereas p53-negative tumors (<10%) exhibited markedly poorer DFS and OS. Differences among the three p53 expression patterns remained statistically significant for both DFS (*p* = 0.00071) and OS (*p* = 0.0094).

Formal interaction analysis supported a differential association betweenp53 expression assessed before NAC and survival according to pathological response. For DFS, p53 expression ≥10% was associated with a lower hazard of recurrence among patients achieving pCR (HR 0.080, 95% CI 0.015–0.411), whereas no significant association was observed among patients with residual disease (HR 1.00, 95% CI 0.35–2.90); the p53-by-pathological-response interaction was significant (*p* interaction = 0.014). A significant interaction was also observed for OS (*p* interaction = 0.015). Because no deaths occurred among the 11 pCR patients with p53 expression ≥10%, Firth penalized Cox regression was used for the OS analysis. Within the pCR subgroup, p53 expression ≥10% was associated with a lower hazard of death (HR 0.047, 95% CI 0.00035–0.443), whereas no significant association was observed among patients with residual disease (HR 0.878, 95% CI 0.303–2.614).

Together, these findings indicate that the association between p53 expression and survival differed according to pathological response, with prognostic stratification observed among patients achieving pCR but not among patients with residual disease. Given the limited number of patients and events in the pCR subgroup, these interaction and subgroup findings should be considered exploratory and require validation in larger independent cohorts. Negative p53 expression (<10%) was associated with increased risk of recurrence and death despite pathological response, whereas intermediate-to-strong p53 expression patterns were associated with more durable disease control and prolonged survival. 

### 3.7. TP53 Variant Landscape and Longitudinal Changes Across Prognostic Groups

**Figure 3:**
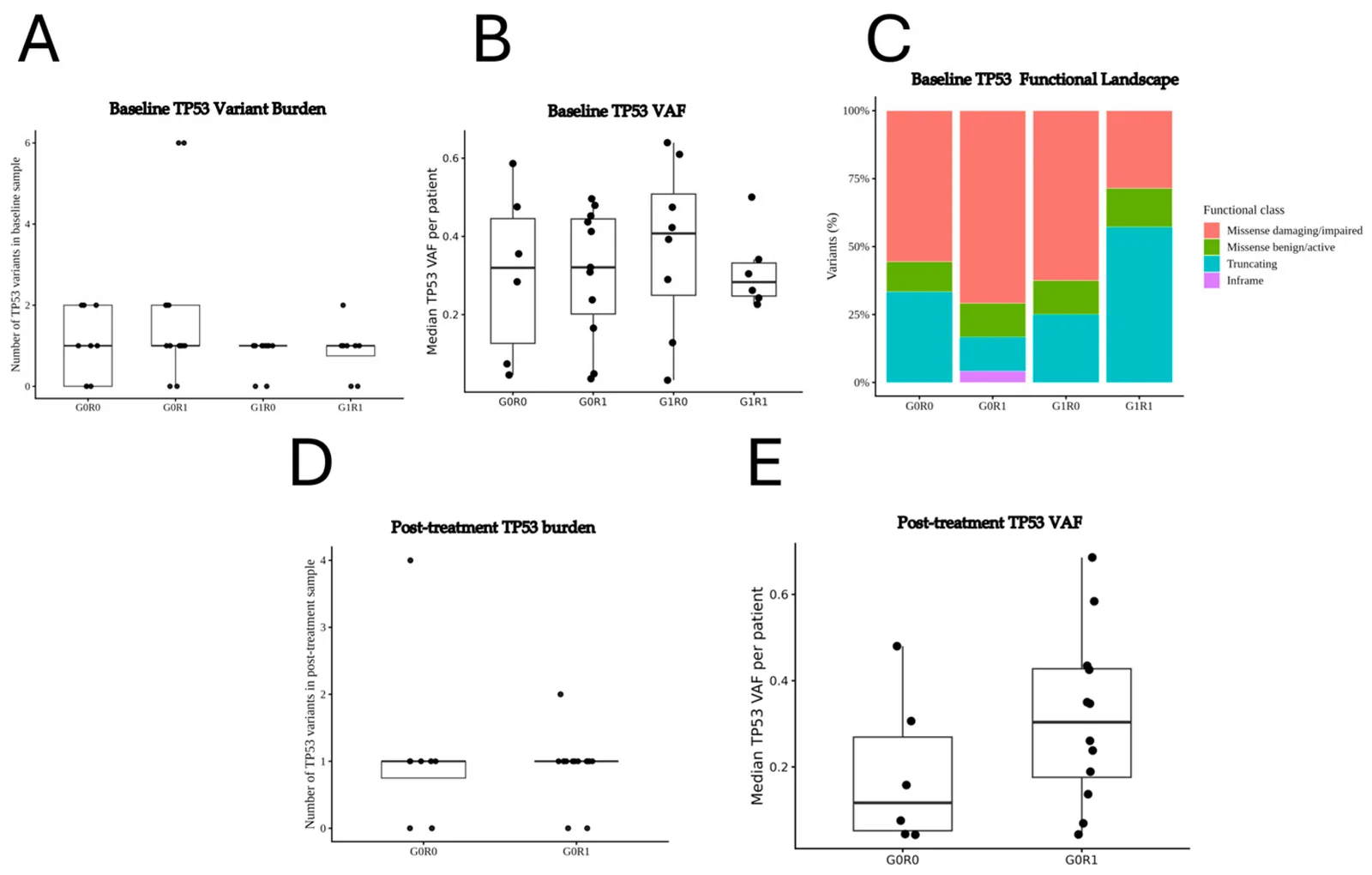
(**A**) Baseline TP53 variant burden across prognostic groups defined by treatment response and recurrence status (G0R0, G0R1, G1R0, and G1R1). Each point represents an individual tumor sample. (**B**) Patient-level median TP53 variant allele frequency (VAF) at baseline across the four prognostic groups. Each point represents one patient. (**C**) Functional classification of baseline TP53 variants according to prognostic group. Variants were categorized as damaging/impaired missense, functionally active missense, truncating, or inframe alterations. (**D**) Post-treatment TP53 **variant burden** in residual tumors from non-responder groups (G0R0 and G0R1). (**E**) Patient-level median TP53 VAF in post-treatment residual tumors from the G0R0 and G0R1 groups. Each point represents one patient.

We next investigated the TP53 molecular landscape at baseline and after treatment according to the combined pathological response and recurrence subgroups (Figure 3). The complete annotated TP53 variant list used for these analyses is provided in Supplementary Table S1.

First, we examined whether p53 immunohistochemical expression before NAC was associated with TP53 variant status. Among the detected TP53 variants, rs1042522 (p.Pro72Arg) was the only variant classified as a population polymorphism and was therefore excluded from the revised molecular analyses. After excluding this polymorphism from TP53 variant-status classification, p53 expression patterns differed significantly between tumors with and without detected TP53 variants. Using the three-group classification, tumors without detected TP53 variants predominantly showed low p53 expression (<10%; 66.7%), whereas tumors with detected TP53 variants were enriched for strong p53 expression (>50%; 63.3%; Fisher’s exact test, *p* = 0.0187). A similar association was observed using the binary IHC classification (<10% vs. ≥10%; *p* = 0.0407). However, TP53 variant status itself was not significantly associated with treatment response, recurrence, DFS, or OS.

At baseline, the overall TP53 variant burden was relatively similar across the four prognostic groups (Figure 3A). Most tumors harbored a limited number of TP53 variants, although G0R1 included the tumors with the highest variant burden observed after filtering. Responders without recurrence (G1R0) and responders with recurrence (G1R1) displayed comparatively narrower distributions. No statistically significant differences in TP53 variant burden were observed among prognostic groups (Kruskal–Wallis *p* = 0.4025).

Analysis of TP53 VAF at the patient level showed broadly overlapping distributions across the four prognostic groups at baseline (Figure 3B). Median patient-level VAFs were 0.320 in G0R0, 0.321 in G0R1, 0.408 in G1R0, and 0.283 in G1R1, with no statistically significant differences among groups (Kruskal–Wallis *p* = 0.8905).

Functional classification of baseline TP53 variants demonstrated that damaging/impaired missense and truncating variants constituted the predominant alteration classes across the prognostic groups (Figure 3C). Damaging/impaired missense variants represented the largest fraction in G0R0, G0R1, and G1R0 tumors, whereas truncating variants represented the largest fraction in G1R1 tumors. Functionally active missense variants constituted a smaller fraction across all groups, and inframe variants were rare and observed only in G0R1 tumors.

We next examined TP53 variants in paired pre- and post-treatment samples from G0 tumors. Post-treatment TP53 variant burden was relatively similar between G0R0 and G0R1 tumors, most samples harboring approximately one detectable TP53 variant after treatment (Figure 3D). Individual tumors showed higher variant burdens, although substantial inter-patient variability was observed, and no statistically significant difference was detected between groups (Wilcoxon *p* = 0.8618). At the patient level, median post-treatment TP53 VAF was 0.117 in G0R0 and 0.304 in G0R1 tumors (Figure 3E). Despite this numerical difference, the distributions substantially overlapped and the difference was not statistically significant (Wilcoxon *p* = 0.2061).

Overall, these findings suggest that neither baseline TP53 variant burden nor patient-level TP53 VAF clearly distinguished the prognostic groups. Differences in functional composition were observed descriptively, but these molecular findings should be interpreted as exploratory.

### 3.8. Longitudinal TP53 Variant Dynamics in Paired Tumor Samples

Baseline sequencing data were available for 40 patients, while post-treatment tumor samples were available for 21 patients. Among these, 18 patients had matched baseline and post-treatment sequencing data and were therefore included in the paired longitudinal analysis.

**Figure 4:**
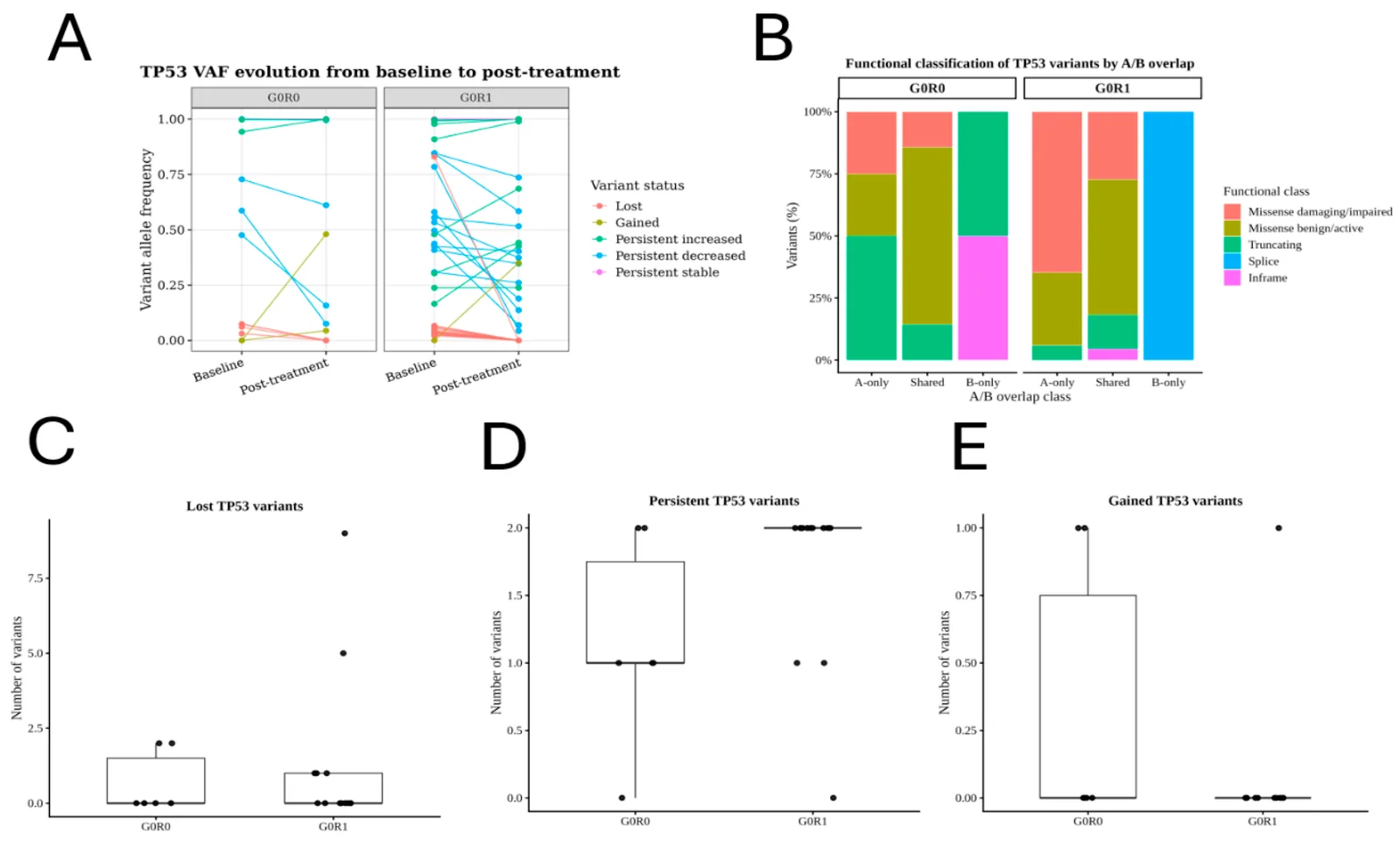
(**A**) Longitudinal changes in TP53 variant allele frequencies (VAF) from baseline to post-treatment in paired tumors with residual disease (G0R0 and G0R1). Variants were classified according to their detection pattern as baseline-only, post-treatment-only, or shared between both timepoints. For shared variants, the direction and magnitude of VAF change between baseline and post-treatment samples are displayed descriptively without applying a categorical threshold for VAF change. (**B**) Functional classification of TP53 variants according to baseline/post-treatment overlap category. Variants were grouped as baseline-only, shared between baseline and post-treatment, or post-treatment-only and categorized according to predicted functional consequences. (**C**–**E**) Quantification of longitudinal TP53 variant patterns in paired tumors with residual disease. (**C**) Number of baseline-only variants per tumor. (**D**) Number of shared variants per tumor. (**E**) Number of post-treatment-only variants per tumor. Each point represents an individual patient.

To further characterize longitudinal changes in TP53 variant detection and VAF, we analyzed paired baseline (a) and post-treatment (b) samples from non-responder tumors (G0 groups) and classified variants according to their longitudinal detection and VAF patterns (Figure 4A).

Most TP53 variants detected after treatment corresponded to variants also detected at baseline. Among variants detected at both timepoints, some showed numerically lower and others numerically higher VAFs in the post-treatment sample. Baseline-only variants were also detected in both groups and varied between patients. These patterns describe differences in variant detection and VAF between paired samples but do not by themselves establish elimination, acquisition, clonal expansion or depletion, or treatment-driven selection.

We next examined the functional composition of variants according to their a/b overlap category (Figure 4B). In G0R0 tumors, variants detected exclusively at baseline (a-only) consisted primarily of truncating and damaging/impaired missense alterations, whereas shared variants were composed of damaging/impaired missense and truncating variants. Interestingly, variants detected only after treatment (b-only) included mainly truncating and in-frame alterations. By contrast, in recurrent non-responder tumors (G0R1), a-only variants were predominantly damaging/impaired missense variants, while shared variants included damaging/impaired missense, truncating, and inframe alterations. Notably, the few b-only variants identified in G0R1 tumors corresponded almost exclusively to splice-related alterations.

Quantitative comparison of longitudinal TP53 variant patterns between G0R0 and G0R1 tumors showed no major differences in the number of baseline-only, shared, or post-treatment-only TP53 variants per tumor (Figure 4C–E). Shared variants were frequently detected in both groups, whereas post-treatment-only variants were uncommon overall and detected only sporadically in individual tumors. Similarly, although some G0R1 tumors exhibited high numbers of baseline-only variants, this pattern was heterogeneous and did not define the entire subgroup.

Finally, we explored whether variants detected in responder tumors overlapped with baseline-only variants or shared variants showing numerically lower post-treatment VAF in paired samples from non-responder/non-recurrent tumors (G0R0). Comparison between baseline variants from responder tumors (G1) and these longitudinal variant patterns in G0R0 tumors identified a small, shared subset of three variants (14% overlap), although the low number of overlapping variants limited further quantitative interpretation. Functional annotation of these shared variants revealed representation of damaging/impaired missense, functionally active missense, and truncating alterations, indicating that variants showing these longitudinal patterns were not restricted to a single functional TP53 class.

Together, these results indicate that TP53 variant profiles differed between baseline and post-treatment samples, with both shared and timepoint-specific variants observed in residual tumors. Shared TP53 variants were common in non-responders, while only a limited subset of variants detected in responder tumors overlapped with baseline-only variants or shared variants showing numerically lower post-treatment VAF in G0R0 tumors. Given the targeted sequencing design and potential effects of tumor purity, sampling, and technical variability on variant detection and VAF, these longitudinal findings were considered descriptive and do not establish clonal evolution or treatment-driven selection.

## 4. Discussion

In this study, we evaluated the prognostic significance of p53 immunohistochemical expression together with the molecular characteristics and longitudinal changes in TP53 variant detection and VAF in TNBC treated with neoadjuvant chemotherapy. The principal clinical finding was that the prognostic association of p53 expression differed according to pathological response, whereas TP53 variant status alone was not significantly associated with treatment response or survival. Among patients achieving pathological complete response (pCR), p53 expression ≥10% was associated with significantly longer disease-free survival (DFS) and overall survival (OS), whereas no comparable prognostic association was observed among patients with residual disease. The three-tier classification further suggested heterogeneity within the pCR subgroup, with intermediate p53 expression (10–50%) showing particularly favorable outcomes. These observations support a context-dependent interpretation of p53 IHC rather than its use as a uniformly favorable or adverse prognostic marker.

The prognostic significance of p53 expression in breast cancer has been investigated for more than three decades, with persistently heterogeneous results. Early studies associated increasing p53 immunoreactivity with higher proliferative activity and poorer clinical outcome. In a large series of node-negative breast cancers, Allred et al. reported progressively shorter DFS with increasing p53 expression and poorer OS among tumors with the highest p53 levels [19]. However, subsequent studies already demonstrated important limitations of interpreting p53 accumulation as a simple surrogate of aggressive tumor biology. Faille et al. observed substantial heterogeneity in the percentage and distribution of p53-positive cells and incomplete correspondence between p53 immunostaining and TP53 alterations in locally advanced breast cancer [20]. Shortly thereafter, Sjögren et al. directly compared p53 IHC with TP53 sequencing and demonstrated considerable discordance between the two approaches; sequencing provided stronger prognostic information than IHC in their cohort [21]. These early observations anticipated an issue that remains relevant today: p53 immunohistochemistry and TP53 genotype are biologically related but are not interchangeable biomarkers.

More recent studies have continued to report on divergent prognostic associations. In TNBC specifically, Li et al. found that p53 positivity was associated with poorer DFS and OS and remained independently prognostic after adjustment for established clinicopathological variables [22]. Conversely, da Silva et al., in a cohort of 171 locally advanced TNBCs treated with neoadjuvant chemotherapy observed no significant association between p53 IHC and either treatment response or survival; residual cancer burden and other post-treatment variables were substantially stronger prognostic determinants [23]. Similarly, recent IHC-based TNBC subtyping studies have not consistently identified p53 expression as an independent survival marker. Together with reports showing associations in the opposite prognostic direction, these findings reinforce the idea that the clinical meaning of p53 IHC may depend strongly on tumor subtype, treatment setting, scoring method, and the population in which it is evaluated [9,10,11].

Our results add another layer of complexity by evaluating p53 expression before treatment in relation to subsequent pathological response. The favorable outcome observed among p53-positive patients achieving pCR should not be interpreted as evidence that increased p53 expression is intrinsically protective. Rather, p53 expression may identify biologically distinct tumors within a group already selected by marked chemotherapy sensitivity. This interpretation is consistent with the established prognostic importance of pCR in TNBC [2,3] and with previous molecular studies showing that the prognostic consequences of TP53 alterations are heterogeneous. Mitri et al. showed that the clinical relevance of TP53 in TNBC depends in part on the functional mutation class rather than on mutation status alone [10]. Similarly, Kim et al., in 174 TNBCs analyzed by p53 IHC, TP53 sequencing, and mRNA expression, demonstrated a strong relationship between mutation type and p53 expression but found that neither mutation class nor protein expression alone uniformly predicted outcome [24]. These observations support the concept that TP53 biology is unlikely to be adequately represented by a single binary molecular or immunohistochemical variable.

A distinctive feature of the present study is the availability of TP53 sequencing data from the same tumor samples obtained before NAC in which p53 IHC was assessed, including tumors that subsequently achieved pCR. Sequencing was performed on tumor tissue without matched normal DNA; therefore, the somatic or germline origin of rare detected variants could not be definitively established. Population-frequency filtering reduced the contribution of common germline polymorphisms but cannot substitute for matched-normal sequencing. After common population polymorphisms were excluded, TP53 variant status was significantly associated with p53 expression: tumors without additional detected TP53 variants were predominantly characterized by low p53 expression, whereas tumors harboring non-polymorphic TP53 variants were enriched for stronger p53 staining. This finding is consistent with both earlier and contemporary studies showing mutation-class-dependent patterns of p53 protein expression. Kim et al. observed high p53 expression particularly in tumors with missense TP53 mutations, whereas deletion-type alterations were associated with substantially lower IHC positivity [24]. More recently, Anderson et al. and Lee et al. confirmed a high, although incomplete, concordance between abnormal p53 IHC patterns and TP53 mutation status, with overexpression predominantly associated with missense alterations and null patterns more frequently associated with truncating variants [8,9,25]. More recently, Kim et al. extended these observations specifically to TNBC, showing that p53 immunostaining patterns were associated not only with TP53 mutation status but also with mutation type and functional characteristics [26]. TP53 mutations are highly prevalent in TNBC, but their prognostic and predictive effects are not uniform and may depend on mutation type, functional consequence, treatment, and residual disease status. Recent experimental evidence further indicates that mutant p53 may actively contribute to TNBC tumor maintenance, emphasizing that the biological consequences of TP53 alterations extend beyond simple mutation status [27]. Importantly, however, TP53 variant status in our cohort was not significantly associated with pathological response, recurrence, DFS, or OS. Thus, the prognostic information captured by p53 IHC cannot be explained simply by the presence or absence of a detectable TP53 variant.

The three-tier analysis provides additional information in this context. Intermediate p53 expression (10–50%) was concentrated in responders without recurrence and was absent among responders who subsequently relapsed. Although this finding is based on a small subgroup and requires independent validation, the concept that intermediate or non-extreme p53 expression may represent a biologically distinct state has precedent. Boyle et al. classified breast cancers into extreme-positive, extreme-negative, and non-extreme p53 immunophenotypes and found poorer OS among tumors showing either extreme pattern compared with the non-extreme group; importantly, the extreme phenotypes also correlated with specific TP53 mutation classes [28]. More recently, Dajti et al. demonstrated that separating null, low/intermediate, and high p53 expression provided prognostic information that could be obscured by a simple binary classification [11]. Although these studies involved different breast cancer populations and scoring systems, they support the broader concept that the relationship between p53 staining, and prognosis is not necessarily linear.

Nevertheless, our three-tier percentage-based classification should not be considered equivalent to contemporary pattern-based approaches that distinguish wild-type, overexpression, null, cytoplasmic, and equivocal phenotypes. Pattern-based assessment may represent a closer pathological surrogate for TP53 functional status. In contrast, our categories were selected principally to evaluate clinically accessible thresholds and to permit comparison with previous prognostic studies. This distinction is important because the historical literature demonstrates that both p53 accumulation and complete absence can correspond to biologically relevant TP53 alterations [20,21,22,23,24,28]. The absence of a universally accepted scoring system therefore remains an important source of heterogeneity among p53 prognostic studies.

At the molecular level, baseline TP53 variant burden did not significantly distinguish the prognostic groups. The use of the term variant burden is deliberate because the tumor-only sequencing design does not allow definitive distinction between somatic and germline variants. Common population polymorphisms, including TP53 p.Pro72Arg, were excluded from the revised molecular analyses. Consequently, the total number of detected variants should not be interpreted as equivalent to pathogenic somatic mutation burden. The absence of prognostic discrimination based solely on variant counts is consistent with the high prevalence and functional heterogeneity of TP53 alterations in TNBC [1,4,10]. Similarly, patient-level TP53 VAF did not significantly differ among prognostic groups at baseline or between G0R0 and G0R1 tumors after treatment. These findings suggest that neither variant burden nor VAF alone provides clear prognostic discrimination in this cohort, while the functional composition and longitudinal patterns of TP53 variants provide additional descriptive molecular context.

The paired analysis of tumor samples obtained before and after NAC provided an additional level of information that could not be captured from the baseline tumor sample alone. In tumors with residual disease, TP53 variants showed heterogeneous patterns between baseline and post-treatment samples, including variants detected at both timepoints, variants detected at only one timepoint, and quantitative differences in variant allele frequency. Many TP53 variants detected after treatment were also present at baseline, frequently with lower but still measurable VAFs, whereas variants detected at only one of the two timepoints were less common. Shared variants with higher post-treatment VAF were also observed. These findings are consistent with molecular heterogeneity between pretreatment and residual tumors after chemotherapy, although changes in variant detection or VAF cannot by themselves establish clonal selection or treatment-driven acquisition or elimination of individual variants. Experimental and clinical studies have similarly demonstrated molecular differences between pretreatment tumors and residual disease following therapy [12,13,14]. In TNBC specifically, Singh et al. reported a broadly stable overall mutational landscape in paired pre- and post-NAC samples while identifying changes in individual alterations [13], and recent genomic and spatial analyses have further demonstrated subtype-specific molecular remodeling of residual breast cancer after treatment [12,15].

The limited overlap between TP53 variants detected only at baseline or showing numerically lower post-treatment VAF in non-responder/non-recurrent tumors further argues against a simple model in which favorable outcome depends on changes in a specific TP53 variant class. Rather, treatment response and long-term prognosis are likely to reflect interactions among TP53 functional status and additional genomic or microenvironmental determinants not captured by the present targeted analysis. In this respect, p53 IHC and TP53 sequencing appear to provide related but non-equivalent information. This observation is consistent with both the earliest direct comparisons of IHC and sequencing [21] and contemporary molecular pathology studies [8,9,24].

Taken together, our findings suggest that p53 IHC may be most informative when interpreted alongside pathological response and molecular context. pCR remains one of the strongest indicators of favorable long-term outcome in TNBC [2,3], but it does not eliminate the risk of recurrence. The observation that p53 expression assessed before NAC treatment further stratified outcomes within the pCR subgroup is therefore potentially relevant, although it should be regarded as hypothesis-generating given the limited number of patients and events. Conversely, among patients with residual disease, the dominant adverse prognostic effect of treatment resistance and the molecular heterogeneity of the remaining tumor may reduce the discriminatory value of a single IHC measurement in tumors before treatment with NAC. The longitudinal sequencing results support this interpretation by demonstrating that the molecular composition of residual disease cannot necessarily be inferred from the baseline tumor alone.

Evidence from independent neoadjuvant breast cancer cohorts further indicates that TP53-related biomarkers may provide clinically relevant information, although different analytical approaches should not be considered interchangeable. TP53 transcriptional signatures have been associated with pCR and prognosis after NAC and validated across independent breast cancer cohorts [29,30]. In contrast, studies evaluating p53 protein expression by IHC in NAC-treated TNBC have reported heterogeneous associations with pathological response and survival [23,24]. These differences may reflect the distinct biological information captured by TP53 genotype, transcriptional phenotype, and p53 protein expression. The present findings extend these observations by examining p53 IHC together with tumor TP53 variant characterization and, importantly, by evaluating the prognostic association of p53 expression according to pathological response.

Several limitations should be considered. This was a retrospective, single-center study with a relatively small cohort, and stratification by pathological response and p53 expression resulted in small patient subgroups with limited statistical power and precision. These subgroup analyses, particularly those restricted to patients achieving pCR, should therefore be considered exploratory and hypothesis-generating rather than confirmatory and require validation in larger, independent cohorts. The limited sample size and number of outcome events also constrained robust multivariable modeling because of the risk of overfitting and unstable effect estimates. Given the limited number of patients carrying any individual TP53 alteration, variant-specific survival analyses were not considered statistically reliable; individual variants and their functional annotations are therefore reported descriptively in Supplementary Table S1. We assessed p53 expression as the percentage of positively stained tumor cells rather than using a fully standardized pattern-based algorithm. In addition, the targeted sequencing panel was not designed to capture the full spectrum of TP53 alterations, including large structural variants, copy-number abnormalities, or regulatory changes. Moreover, sequencing was performed on tumor tissue without matched normal samples; therefore, detected TP53 variants could not be definitively classified as somatic or germline. Longitudinal differences in variant detection and VAF may also be influenced by tumor cellularity, copy-number alterations, spatial sampling, sequencing depth, and technical variability and should not be interpreted as direct evidence of clonal acquisition, elimination, expansion, depletion, or treatment-driven selection. Post-treatment tumor material was necessarily unavailable in patients achieving pCR, restricting longitudinal sequencing to tumors with residual disease. Finally, the cohort was treated before the routine incorporation of contemporary neoadjuvant chemoimmunotherapy and current post-neoadjuvant strategies, which should be considered when extrapolating these observations to present-day clinical practice. Nevertheless, the availability of p53 IHC and TP53 sequencing in the same tumors before NAC treatment, together with paired longitudinal molecular data in residual disease, represents an important strength of the study.

## 5. Conclusions

Our results support a context-dependent prognostic role for p53 immunohistochemistry in TNBC treated with neoadjuvant chemotherapy. p53 expression assessed before NAC provided prognostic stratification primarily among patients achieving pCR, whereas TP53 variant status and baseline variant burden alone did not show comparable prognostic discrimination. Longitudinal sequencing additionally revealed shared variants and changes in the detection and relative abundance of TP53 variants between baseline and post-NAC residual disease. Overall, these findings suggest that the prognostic significance of p53 immunohistochemical expression may depend on pathological response, whereas TP53 sequencing provides descriptive molecular information on longitudinal tumor changes rather than independent prognostic stratification. Larger independent cohorts using standardized p53 pattern assessment and contemporary treatment regimens will be required to validate these observations.

## Data Availability

The datasets generated and analyzed during the current study are available from the corresponding and first author upon request.

## Supplementary Materials

The following supporting information can be downloaded at: https://www.mdpi.com/article/10.3390/diagnostics16193229/s1, Table S1: Annotated TP53 variants identified in pretreatment and post-treatment tumor samples; Table S2: Demographic, clinicopathological, treatment-related, and molecular characteristics of the study cohort. Figure S1: Patient and sample flow according to data availability and analysis population.

## Abbreviations

The following abbreviations are used in this manuscript:

Abbreviation	Definition
AJCC	American Joint Committee on Cancer
DFS	Disease-free survival
ER	Estrogen receptor
FFPE	Formalin-fixed paraffin-embedded
HER2	Human epidermal growth factor receptor 2
IHC	Immunohistochemistry
NAC	Neoadjuvant chemotherapy
OS	Overall survival
Pcr	Pathological complete response
PR	Progesterone receptor
TNBC	Triple-negative breast cancer
VAF	Variant allele frequency
VEP	Variant Effect Predictor
