# Supplementary material for: Prognostic Significance of p53 Immunohistochemical Expression and Molecular Characterization of TP53 Variants in Triple-Negative Breast Cancer After Neoadjuvant Chemotherapy
Source: Diagnostics (Basel). 2026 Oct 6;16(19):3229. doi: 10.3390/diagnostics16193229 (PMC13663948; doi:10.3390/diagnostics16193229)
Supplement: Supplementary file 1 [file diagnostics-16-03229-s001.zip › diagnostics-4546412-supplementary/Supplementary Figure 1.pdf]

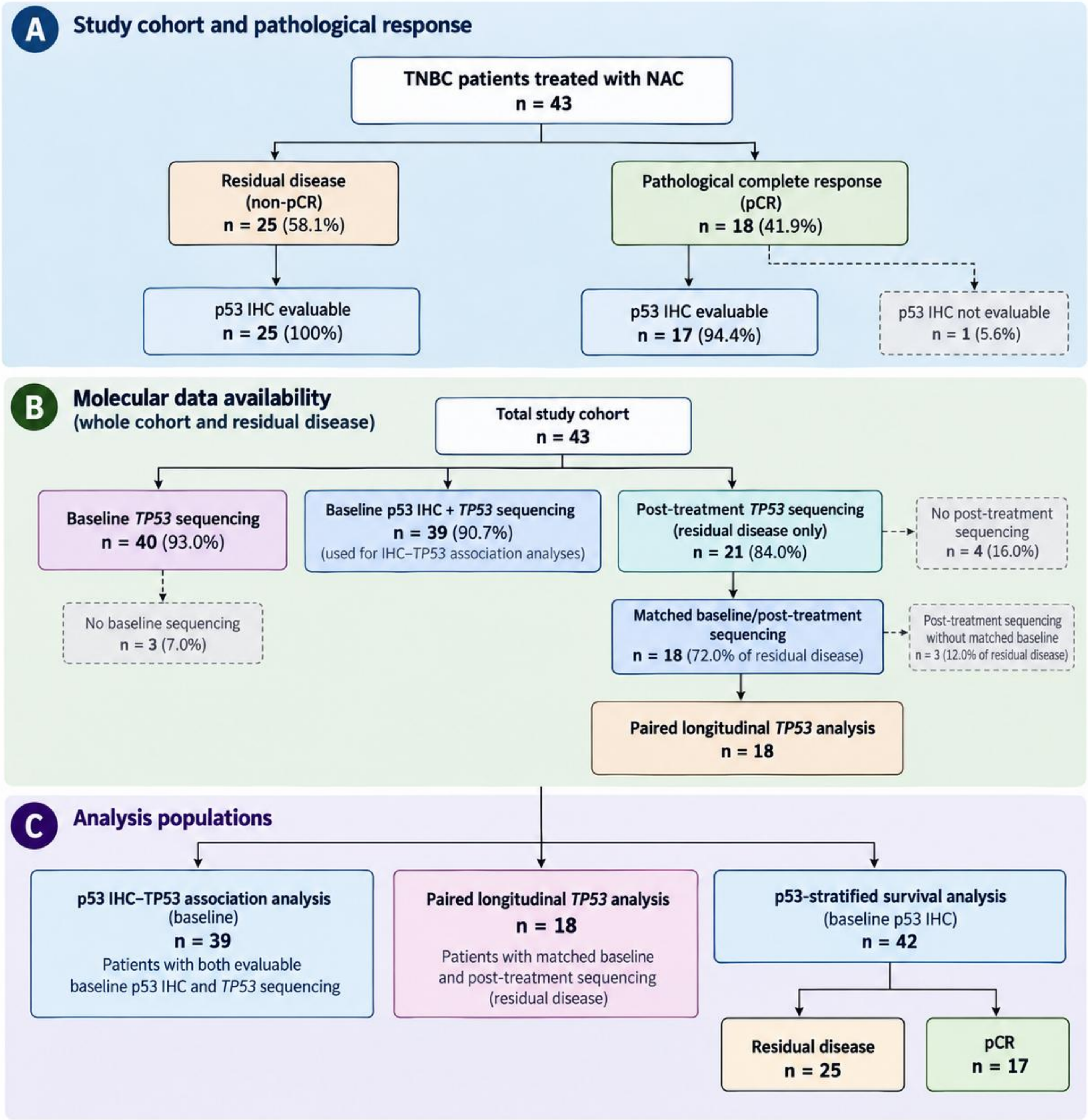

Supplementary Figure S1. Patient and sample flow according to data availability and analysis population. The complete cohort comprised 43 patients, including 25 with residual disease and 18 who achieved pathological complete response (pCR). Baseline p53 immunohistochemistry (IHC) was evaluable in 42 patients, including 17 patients with pCR. Baseline TP53 sequencing was available for 40 patients, and 39 patients had both evaluable baseline p53 IHC and TP53 sequencing. Post-treatment TP53 sequencing was available for 21 patients with residual disease, of whom 18 had matched baseline and post-treatment sequencing data and were included in the paired longitudinal analysis. p53-stratified survival analyses included the 42 patients with evaluable baseline p53 IHC
