# Supplementary material for: Prognostic Significance of p53 Immunohistochemical Expression and Molecular Characterization of TP53 Variants in Triple-Negative Breast Cancer After Neoadjuvant Chemotherapy
Source: Diagnostics (Basel). 2026 Oct 6;16(19):3229. doi: 10.3390/diagnostics16193229 (PMC13663948; doi:10.3390/diagnostics16193229)
Supplement: Supplementary file 1 [file diagnostics-16-03229-s001.zip › diagnostics-4546412-supplementary/Supplementary table S2.pdf]

**Supplementary Table S2. Demographic, clinicopathological, treatment-related, and outcome characteristics of the study cohort (n = 43).**

| Variable | Value |
|---|---|
| **Patients, n** | 43 |
| **Age at diagnosis, median (IQR), years** | 55 (45–67) |
| **Breast side** | Left: 18 (41.9%); Right: 25 (58.1%) |
| **Family history of breast cancer** | 10 (23.3%) |
| **Age at menarche, median (IQR), years** | 13 (12–13.5) |
| **Clinical stage** | Stage I: 2 (4.7%); Stage II: 32 (74.4%); Stage III: 9 (20.9%) |
| **Clinical T stage** | T1: 11 (25.6%); T2: 28 (65.1%); T3–T4: 4 (9.3%) |
| **Clinical nodal involvement** | 21 (48.8%) |
| **Histological subtype** | Invasive ductal carcinoma: 40 (93.0%); Other: 3 (7.0%) |
| **Histological grade** | Grade 2: 12 (27.9%); Grade 3: 31 (72.1%) |
| **Nuclear pleomorphism** | Moderate/Marked: 32 (74.4%) |
| **Ki67 ≥ 14%** | 36 (83.7%) |
| **Neoadjuvant chemotherapy cycles, median (IQR)** | 6 (6–8) |
| **Pathological complete response (pCR)** | 18 (41.9%) |
| **Residual disease** | 25 (58.1%) |
| **Breast-conserving surgery** | 26 (60.5%) |
| **Disease recurrence** | 22 (51.2%) |
| **Deaths during follow-up** | 17 (39.5%) |
| **Disease-free survival, median (IQR), months** | 58.4 (22.7–97.5) |
| **Overall survival, median (IQR), months** | 69.7 (39.6–106.8) |
